# Using Stories to Integrate Gerontology Content Into an Introductory Design (Architecture) Course

**DOI:** 10.1093/geroni/igaa057.004

**Published:** 2020-12-16

**Authors:** Gail Towsley, Jim Agutter, Antonius Tsai, Jareth Archer, Sarah Sovereen, Molly Hanrahan

**Affiliations:** University of Utah, Salt Lake City, Utah, United States

## Abstract

We describe how a partnership between design and gerontology introduces students to concepts of legacy, meaning making and empathy in an undergraduate design course. Introduction to Design Thinking is offered through the Multi-disciplinary Design Program at the University of Utah and is a class that explores concepts such as the design process, human-centered design, rapid prototyping and multidisciplinary team dynamics. In collaboration with faculty from design, medicine and gerontology, the course addresses real-world problems by navigating across disciplines such as art, business, engineering, science, and gerontology. Five phases of the design process are introduced: Observation (collecting material), Analysis (finding patterns and insight), Ideation (solution exploration), Refinement, and Implementation (communication). To apply these processes, students engage in a group project where they interact with older adults residing in long term care (e.g., assisted living). Students undergo HIPAA training and didactic lectures covering narrative interviewing and aging content such as long term care and generativity prepare students for interacting with residents. Throughout the semester, students meet with the older adult to understand, gather, construct and then develop a designed artifact that reflects the stories and the memories of the participating residents. The designed artifacts (e.g., memory maps, videos, small books) are presented and given to the participating residents and their families. This project results in rich stories for both the residents and students. Students also gain interviewing and empathy skills necessary for designing compelling artifacts and potentially age-friendly long term care environments where 2 million older adults live.

